# Typification of names in *Kaempferia* (Zingiberaceae) in the flora of Cambodia, Laos and Vietnam

**DOI:** 10.3897/phytokeys.122.32160

**Published:** 2019-05-28

**Authors:** Oudomphone Insisiengmay, Thomas Haevermans, Mark F. Newman

**Affiliations:** 1 Sorbonne Université Paris France; 2 Ministry of Science and Technology Vientiane Lao People's Democratic Republic; 3 Royal Botanic Garden Edinburgh Edinburgh United Kingdom

**Keywords:** *
Kaempferia
*, nomenclature, Lao PDR, typification

## Abstract

Neotypes are designated for five names in *Kaempferia* (Zingiberaceae) from Lao PDR, namely *K.attapeuensis* Picheans. & Koonterm, *K.champasakensis* Picheans. & Koonterm, *K.gigantiphylla* Picheans. & Koonterm, *K.sawanensis* Picheans. & Koonterm and *K.xiengkhouangensis* Picheans. & Phokham.

## Introduction

*Kaempferia* L. is a genus of ca. 40 species distributed in tropical Asia (Mabberley 2017). Gagnepain (1908: 45–54) recognised 13 species of *Kaempferia* in Cambodia, Laos and Vietnam, nine of which are still placed in this genus. The Thai Forest Bulletin (Botany) by Sirirugsa (1992: 1–15) recorded 15 species in Thailand and 10 species in Cambodia, Laos and Vietnam (Sirirugsa 1992).

Twelve names were coined by C. Picheansoonthon and his colleagues from 2008–2013, namely *Kaempferiaattapeuensis* Picheans. & Koonterm (2009b: 221), *Kaempferiachampasakensis* Picheans. & Koonterm (2008: 406), *Kaempferiagigantiphylla* Picheans. & Koonterm (2009b: 219), *Kaempferiakoratensis* Picheans. (2011: 4), *Kaempferiakoontermii* Prasarn, Wongsuwan & Picheans. (Wongsuwan et al. 2015: 29), *Kaempferialopburiensis* Picheans. (2010: 148), *Kaempferiapicheansoonthonii* Wongsuwan & Phokham (Phokham et al. 2013: 302), *Kaempferiasaraburiensis* Picheans. (2011: 1), *Kaempferiasawanensis* Picheans. & Koonterm (2009: 509), *Kaempferiasisaketensis* Picheans. & Koonterm (2009a: 52), *Kaempferiaudonensis* Picheans. & Phokham (Phokham et al. 2013: 299) and *Kaempferiaxiengkhouangensis* Picheans. & Phokham (Phokham et al. 2013: 305). Five of these species are based on type specimens collected in Laos and are being studied and cited in a revision of *Kaempferia* for the *Flora of Cambodia, Laos and Vietnam* currently in preparation.

The protologues of *Kaempferiaattapeuensis*, *K.champasakensis*, *K.gigantiphylla*, *K.sawanensis* and *K.xiengkhouangensis* state that the type specimens were collected in Laos and deposited at BK, BKF and SING. Paratypes of *Kaempferiaattapeuensis*, *K.gigantiphylla* and *K.xiengkhouangensis* were also cited at the same herbaria. In order to locate this material, the first author visited BK, BKF and SING but, at each of these herbaria, no type specimens or paratype specimens were found. Attempts were made to contact the authors of these names and to encourage them to deposit the specimens cited in their protologues. No reply was received, either to our enquiries or to those of the curator of BKF so we are obliged to review the original material of each name and, as necessary, to select neotypes to replace the missing holotypes and isotypes. If the holotypes and isotypes are found to exist in the future, then the neotypifications made below will be superseded (Turland et al. 2018, Art. 9.19).

## Materials and methods

The type locality of each name was visited and a search was made for plants which matched the protologue. In some cases, the type locality was clearly indicated by GPS coordinates but we also found cases in which the GPS coordinates appear to be in error, as noted below.

## Result

### Neotypifications

#### Kaempferia attapeuensis

Taxon classificationPlantaeZingiberalesZingiberaceae

Picheans. & Koonterm, Taiwania 54(3): 221. 2009.

##### Type.

 Lao PDR, Attapeu Province, Ban Oudomxay, 14°45.167'N, 106°60.415'E (*sic*), alt. 80 m, 5 Jul 2007, *C. Picheansoonthon & S. Koonterm 123* (holotype BKF; isotypes: BK, SING). Lao PDR, Attapeu Province, Xaysettha District, Ban Touy, 14°50.305'N, 106°57.51'E, 113 m alt., 20 Jun 2016, *O. Insisiengmay OI-141* (neotype: HNL, designated here; isoneotypes: E (with spirit collection), P (with spirit collection)).

##### Note.

 The original material comprises the type and one further collection, *Picheansoonthon & Koonterm 229* (BKF, *n.v.*), from Ban Kasome, Attapeu Province, 14°50.167'N, 106°57.515'E, alt. 88 m, 5 Jul 2008. The GPS coordinates of the holotype locality are in error (there are only 60 minutes in a degree so the longitude given is impossible) and, furthermore, Ban Oudomxay cannot be traced in Attapeu. The paratype locality, Ban Kasome, exists and a small collection (*OI-146*) matching the protologue was made there in June 2016. A larger, identical collection (*OI-141*) was made at a nearby locality and it is this which is designated here as neotype.

#### Kaempferia champasakensis

Taxon classificationPlantaeZingiberalesZingiberaceae

Picheans. & Koonterm, Taiwania 53(4): 406. 2008.

##### Type.

 Lao PDR, Champasak Province, Xanasomboun District, Ban Lat Suea, 15°18.954'N, 105°38.654'E, alt. 109 m, 10 Jun 2007, *C. Picheansoonthon & S. Koonterm 52* (holotype: BKF; isotypes: BK, SING). Lao PDR, Champasak Province, Xanasomboun district, Boung Kha village, Ya Nang stream, 15°18.955'N, 105°38.5933'E, 105 m alt., 19 Jun 2016, *O. Insisiengmay OI-128* (neotype: HNL, designated here; isoneotypes: BKF, E (with spirit collection), P (with spirit collection), SING).

##### Note.

 This species is based on the type collection alone. No paratype material was cited in the protologue. The type locality was found using the GPS coordinates given in the protologue though the nearby village is called Ban Boung Kha, not Ban Lat Suea. A collection, *O. Insisiengmay OI-128*, was made at this location and it matches the description in the protologue exactly. This is designated here as the neotype of *Kaempferiachampasakensis*.

#### Kaempferia gigantiphylla

Taxon classificationPlantaeZingiberalesZingiberaceae

Picheans. & Koonterm, Taiwania 54(3): 219. 2009.

##### Type.

Lao PDR, Salawan Province, Tad Loa Waterfalls, 15°27.363'N, 106°44.12'E, alt. 482 m, 4 Jul 2007, *C. Picheansoonthon & S. Koonterm 117* (holotype BKF; isotypes: BK, SING). Lao PDR, Salawan Province, Tad Lo Waterfalls, 15°31.6633'N, 106°16.535'E, 370 m alt., 21 Jun 2016, *O. Insisiengmay OI-149* (neotype: HNL, designated here; isoneotypes: E (with spirit collection), P (with spirit collection)).

##### Note.

 The original material comprises the type and two paratype collections, as follows: Champasak Province, Phou Savan, 15°23.355'N, 105°41.318'E, alt. 696 m, 14 Jun 2007, *Picheansoonthon & Koonterm 105* (BK, *n.v.*); Attapeu Province, Ban Kasome, 14°50.167'N, 106°57.515'E, alt. 88 m, 26 Apr 2008, *Picheansoonthon & Koonterm 219* (BK, *n.v.*). The type locality is a well-known place which is easy to find. The neotype collection was made at Tad Lo and matches the protologue exactly.

#### Kaempferia sawanensis

Taxon classificationPlantaeZingiberalesZingiberaceae

Picheans. & Koonterm, Acta Bot. Yunnan. 31(6): 509. 2009

##### Type.

 Lao PDR, Sawanakhet province, Phin District, Dong Phou Vieng NPA, 16°30.457'N, 106°1.446'E, alt. 288 m, 26 Apr 2007, *C. Picheansoonthon & S. Koonterm 16* (holotype: BKF; isotypes: BK, SING). Lao PDR, Sawanakhet Province, Phin District, Dong Phou Vieng NBCA, 16°25.385'N, 105°58.6217'E, 289 m alt., 7 Jun 2016, *O. Insisiengmay OI-49* (neotype: HNL, designated here; isoneotypes: BKF, E (with spirit collection), FOF, P (with spirit collection), SING).

##### Note.

The protologue cites only the type collection, comprising the holotype and two isotypes. No other collections were cited as paratypes so the existing original material consists of the published text and figures alone.

A search for *Kaempferiasawanensis* was made at the type locality, using the GPS coordinates given in the protologue. These coordinates lead to an area of rice fields with patches of heavily disturbed forest near human habitation just south of the district town of Phin. No plants which matched the protologue could be found in this area.

Proceeding southwards along the same road, Dong Phou Vieng NPA is reached as the land begins to rise. After a right turn, the road leads towards Ban Tad Hai through evergreen forest with clearings. *Kaempferiasawanensis* was found in one of these clearings.

In the protologue, the habitat is described as “sandy soil along dry evergreen forest and pine-deciduous dipterocarp forest”. Along the road to Ban Tad Hai, a substantial population of *Kaempferiasawanensis* was found in deciduous forest with bamboo (*Vietnamosasa* sp.) but completely without pine. The collectors of the holotype may have misunderstood the name for *Vietnamosasa* bamboo, which is *kok phêk* or *ya phêk* in Laotian, very close to *kok pek* (*Pinus* sp.). In any case, this locality matches the habitat described in the protologue and our collection, *O. Insisiengmay OI-49* matches the description of *Kaempferiasawanensis*. We designate this collection as the neotype.

#### Kaempferia xiengkhouangensis

Taxon classificationPlantaeZingiberalesZingiberaceae

Picheans. & Phokham, J. Jap. Bot. 88: 305. 2013.

##### Type.

 Lao PDR, Xiengkhouang District, Mueang Kham, 19°33.139'N, 103°44.384'E, alt. 600–720 m, 25 Mar 2011, *C. Picheansoonthon & S. Phokham 250311-1* (holotype: BKF; isotypes: BK, SING). Lao PDR, Xiengkhouang Province, Kham District, Houay Phad village, 19°32.6383'N, 103°45.1917'E, 700 m alt., 29 Apr 2016, *O. Insisiengmay OI-2* (neotype: P, designated here).

##### Note.

 The original material cited in the protologue comprises the type collection and a paratype, *Picheansoonthon & Phokham 150711-1* (BKF, *n.v.*), collected in the same place as the type, on 15 Jul 2011. The first author travelled towards the GPS coordinates given in the protologue, stopping at the nearest village to ask whether anyone remembered the Thai collectors who had visited the area in 2011. The villagers did remember and were able to give directions to the type locality. *O. Insisiengmay OI-2* was collected at the type locality and matches the description in the protologue. It is designated here as the neotype.

### A note on *Kaempferiachayanii*

#### Kaempferia chayanii

Taxon classificationPlantaeZingiberalesZingiberaceae

Koonterm, Folia Malaysiana 9(1): 19. 2008.

##### Type.

 Lao PDR, Champasak Province, Xanasomboon Town, Baan Kamphaeng, 15°22.548'N, 105°45.577'E, alt. 610 m, 10 Jun 2007, *C. Picheansoonthon, & S. Koonterm 021* (holotype: BKF; isotypes: BK, SING).

##### Note.

 This species belongs in KaempferiasubgenusKaempferia. As in the cases above, the type material cited in the protologue cannot be found at BK, BKF or SING so a search of the type locality in Champasak Province was made. No material was found to match the protologue, so this species remains to be neotypified.

## Supplementary Material

XML Treatment for Kaempferia attapeuensis

XML Treatment for Kaempferia champasakensis

XML Treatment for Kaempferia gigantiphylla

XML Treatment for Kaempferia sawanensis

XML Treatment for Kaempferia xiengkhouangensis

XML Treatment for Kaempferia chayanii
